# Transcriptome and chromatin accessibility in porcine intestinal epithelial cells upon Zearalenone exposure

**DOI:** 10.1038/s41597-019-0313-1

**Published:** 2019-12-03

**Authors:** Haifei Wang, Jian Jin, Jiayun Wu, Huan Qu, Shenglong Wu, Wenbin Bao

**Affiliations:** 1 Key Laboratory for Animal Genetics, Breeding, Reproduction and Molecular Design, College of Animal Science and Technology, Yangzhou University, Yangzhou, 225009 China; 2 Joint International Research Laboratory of Agriculture & Agri-Product Safety, Yangzhou University, Jiangsu, Yangzhou 225009 China

**Keywords:** Non-coding RNAs, Chromatin

## Abstract

Zearalenone (ZEA) is one of the main mycotoxins widely spread in contaminated cereal crops, which poses a great threat to food safety as well as human and animal health. Biological control strategies are emerging as important solutions to eliminate mycotoxin contaminations. However, molecular mechanisms underlying ZEA cytotoxic effects are only partly understood. Noncoding RNAs and chromatin accessibilities are important regulators of gene expression and implicate in a variety of biological processes. Here, we established a study model of porcine intestinal epithelial cells upon ZEA exposure and presented a RNA-seq dataset for mRNA, microRNA, and lncRNA profiling in 18 experimental samples. In addition, chromatin accessibilities of four samples were also explored by ATAC-seq. This dataset will shed new light on gene expression profiling and transcriptional regulation of animal cells in the response to ZEA exposure, which further contributes to detecting biomarkers and drug targets for predicting and controlling ZEA contamination.

## Abstract (summary)

**Table:** 

Measurement(s)	chromatin organization involved in regulation of transcription • mRNA • lnc_RNA • miRNA
Technology Type(s)	assay for transposase-accessible chromatin using sequencing • RNA sequencing
Factor Type(s)	zearalenone exposure
Sample Characteristic - Organism	Sus scrofa
Sample Characteristic - Environment	cereal grain food product • intestinal epithelium

Machine-accessible metadata file describing the reported data: 10.6084/m9.figshare.10272203

## Background & Summary

Zearalenone (ZEA) is one the main mycotoxins produced by a variety of Fusarium fungal species and widely spread in contaminated cereal crops including maize, wheat, barley and oats^1^. After ingestion and absorption, ZEA is mainly metabolized by intestinal cells and hepatocytes. Because of the structural similarities of ZEA to endogenous estrogen, ZEA can result in serious endocrine disruption and reproductive disorders in animals^2,3^. In addition, ZEA was also found to cause toxic effects on liver and kidney functions^4,5^, and lymphocyte proliferation^6^. ZEA is chemically stable and cannot be removed by the manufacturing process, which poses great risks to food safety as well as human and animal health. Biological control strategies are emerging as promising solutions to eliminate mycotoxin contaminations. Therefore, it is becoming increasingly important to further understand the molecular mechanisms underlying ZEA toxic effects for developing strategies controlling ZEA contamination. Disruption of gene expression programs is an important event through which mycotoxins exert cytotoxic effects. Recent studies have preliminarily investigated the effects of ZEA exposure on genome wide gene expression in porcine epithelial cells^7,8^. However, the regulatory networks involved in gene expression alterations in animal cells upon ZEA exposure remain largely unknown.

Long non-coding RNAs (lncRNAs) and microRNAs (miRNAs) are noncoding RNAs that act as important regulators involved in a variety of physiological, developmental and disease processes at the post-transcriptional level of their target genes^9^. LncRNAs are a class of transcripts with the length of greater than 200 nucleotides, and miRNAs are transcripts with the length of ~22 nucleotides. LncRNAs and miRNAs can either independently regulate target mRNA expression or functionally interact to control the expression of target mRNAs^10^. Therefore, identification of expression patterns of lncRNAs and miRNAs can greatly contribute to revealing the molecular events relevant to the phenotypic changes. Chromatin accessibility represents genomic regions binding with regulatory factors responsible for gene transcription, which can be measured by Tn5 transposase-accessible chromatin sequencing (ATAC-seq). Recently, ATAC-seq has become an effective and powerful tool to capture open chromatin to identify the regulatory elements of gene transcription^11^.

**Fig. 1:**
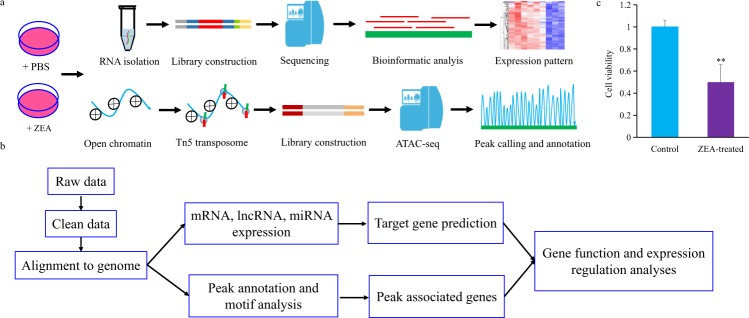
Overview of study design and data analysis workflow. (**a**) Collection and preparation of experimental samples. (**b**) The data analysis workflow for sequencing data. (**c**) Effects of ZEA exposure on cell viability of porcine intestinal epithelial cells IPEC-J2. Bars indicate mean ± standard deviation (n = 3). **P < 0.01.

In this study, we performed genome-wide analyses of the expressions of mRNA, miRNA, and lncRNA in porcine intestinal epithelial cells upon ZEA exposure (Fig. 1a). In total, 18 samples were sequenced on the Illumina Hiseq Platform, generating a total of 1,052,122,031 clean reads after quality control (Tables 1, 2). In addition, changes in chromatin accessibilities upon ZEA exposure were also explored by ATAC-seq (Fig. 1a; Table 1), which yielded a total of 230,639,896 clean reads (Table 3). Integrative bioinformatic analysis workflow of RNA-seq and ATAC-seq data is shown in Fig. 1b. These data will provide comprehensive insight into gene expression profiles and transcriptional regulation of animal cells in the response to ZEA exposure, which may aid the detection of biomarkers and drug targets for predicting and controlling ZEA contamination.

**Table 1:** Overview of experimental samples and sequencing strategy.

Group	Sample ID	Sequencing strategy
ZEA treatment	mZEAT1, mZEAT2, mZEAT3	mRNA sequencing
	miZEAT1, miZEAT2, miZEAT3	microRNA sequencing
	lncZEAT1, lncZEAT2, lncZEAT3	lncRNA sequencing
	atacZEAT1, atacZEAT2	ATAC sequencing
Control	mCTR1, mCTR2, mCTR3	mRNA sequencing
	miCTR1, miCTR2, miCTR3	microRNA sequencing
	lncCTR1, lncCTR2, lncCTR3	lncRNA sequencing
	atacCTR1, atacCTR2	ATAC sequencing

**Table 2:** Summary statistics for mRNA, miRNA, and lncRNA sequencing data.

Sample ID	Raw reads	Clean reads (%)	Q20 (%)	Q30 (%)	GC content (%)	Total mapped (%)	Accession
mZEAT1	65442452	64092924 (97.94)	97.59	93.64	53.82	60140448 (93.83)	SRR9945711
mZEAT2	49134000	47847138 (97.38)	97.47	93.41	53.64	44644114 (93.31)	SRR9945710
mZEAT3	53309044	52070580 (97.68)	97.64	93.79	53.35	48811296 (93.74)	SRR9945713
mCTR1	49591798	48587854 (97.97)	97.66	93.79	53.67	45639499 (93.93)	SRR9945712
mCTR2	49852674	48856876 (98.00)	97.74	94.00	54.13	45976517 (94.1)	SRR9945715
mCTR3	46269648	45523266 (98.39)	97.66	93.39	54.43	42748329 (93.9)	SRR9945714
miZEAT1	12056083	11894381 (98.66)	99.67	98.98	47.72	10896824 (91.61)	SRR9945717
miZEAT2	15501044	15294791 (98.67)	99.72	99.17	47.72	14019486 (91.66)	SRR9945716
miZEAT3	14583982	14459863 (99.15)	99.75	99.25	47.49	13437455 (92.93)	SRR9945719
miCTR1	14187930	13919786 (98.11)	99.63	98.89	47.87	12768598 (91.73)	SRR9945718
miCTR2	14263250	13996174 (98.13)	99.52	98.37	47.92	12655611 (90.42)	SRR9945705
miCTR3	14047502	13864336 (98.70)	99.66	98.97	47.66	12765372 (92.07)	SRR9945704
lncZEAT1	118815840	117197900 (98.64)	96.54	91.06	49.13	109390134 (93.34)	SRR9945703
lncZEAT2	105077302	103377510 (98.38)	96.96	91.87	47.64	97397851 (94.22)	SRR9945702
lncZEAT3	125102564	123413498 (98.65)	96.15	90.33	47.55	114276029 (92.6)	SRR9945709
lncCTR1	104492388	103043178 (98.61)	96.6	91.18	46.39	96304905 (93.46)	SRR9945708
lncCTR2	104128470	102438008 (98.38)	97.67	93.52	47.46	95792468 (93.51%)	SRR9945707
lncCTR3	114192594	112243968 (98.29)	96.57	91.18	47.77	102933819 (91.71)	SRR9945706

**Table 3:** Summary statistics for ATAC-seq data.

Sample ID	Raw reads	Clean reads (%)	Q20 (%)	Q30 (%)	Non-mitochondrial mapped (%)	Accession
atacZEAT1	71215928	71214222 (99.99)	98.88	95.99	61560564 (86.44)	SRR9945701
atacZEAT2	53119698	53112547 (99.99)	98.51	95.11	46843372 (88.20)	SRR9945700
atacCTR1	61879862	61879361 (99.99)	98.69	95.49	49919250 (80.67)	SRR9945720
atacCTR2	44434012	44433766 (99.99)	97.66	93.02	35316004 (79.48)	SRR9945721

## Methods

### Sample preparation and collection

Porcine intestinal epithelial cells (IPEC-J2) were inoculated in 6-well plate at a density of 5 × 10^5^ cells/mL and cultured overnight in a CO_2_ incubator at 37 °C. ZEA was then added to the medium of experimental wells at a final concentration of 10 µg/mL, which can induce cytotoxicity in porcine intestinal epithelial cells as previously reported^12,13^. An equal volume of phosphate buffer saline was added to the medium of control wells. ZEA-treated and control cells were cultured for 48 h and collected for RNA-seq and ATAC-seq (Table 1). Cell viability was gauged using the Cell Counting Kit-8 following the manufacturer’s protocols (Dojindo, Kumamoto, Japan) on the platform of Tecan Infinite 200 microplate reader (Sunrise, Tecan, Switzerland). Significant reduction of cell viability was observed upon ZEA exposure, indicating the toxic effects elicited by ZEA on IPEC-J2 cells (Fig. 1c). Three ZEA-treated and three control samples were collected for mRNA, microRNA, and lncRNA sequencing, respectively (Table 1). In addition, two ZEA-treated and two control samples were collected for ATAC-seq analysis (Table 1).

### Library preparation for mRNA sequencing

Total RNA of the experimental samples was extracted using the Trizol method following the manufacturer’s protocols (Tiangen, Beijing, China). A total amount of 3 µg of RNA per sample was used for mRNA sequencing library preparations using NEBNext Ultra RNA Library Prep Kit for Illumina (NEB, MA, USA) following the manufacturer’s protocols. Index codes were added to attribute sequences to each sample. The library was quantified using the Qubit 2.0 Fluorometer (Thermo Scientific, MA, USA) and diluted into 1 ng/μL, and the library quality was assessed on the Agilent Bioanalyzer 2100 system (Agilent Technologies, CA, USA). Clustering of the index-coded samples was conducted on a cBot Cluster Generation System using TruSeq SR Cluster Kit v3-cBot-HS (Illumia, CA, USA) according to the manufacturer’s guidelines. Following cluster generation, the library preparations were then sequenced on the Illumina Hiseq. 2500 platform and 150 bp paired-end reads were yielded.

### Library preparation for miRNA sequencing

Total RNA of the experimental samples was extracted using the Trizol method following the manufacturer’s protocols (Tiangen, Beijing, China). A total amount of 3 μg of RNA for each sample was used for sequencing library preparation using the NEBNext Small RNA Library Prep Set for Illumina (NEB, MA, USA) following the vendor’s instructions (Illumina, CA, USA). The library was quantified using the Qubit 2.0 Fluorometer (Thermo Scientific, MA, USA) and diluted into 1 ng/μL, and the library quality was assessed on the Agilent Bioanalyzer 2100 system (Agilent Technologies, CA, USA) using DNA High Sensitivity Chips. Clustering of the index-coded samples was conducted on a cBot Cluster Generation System using TruSeq SR Cluster Kit v3-cBot-HS (Illumina, CA, USA) according to the manufacturer’s guidelines. The library preparations were then sequenced on the Illumina Hiseq. 2500 platform and 50 bp single-end reads were produced.

### Library preparation for lncRNA sequencing

Total RNA of the experimental samples was extracted using the Trizol method following the manufacturer’s protocols (Tiangen, Beijing, China). A total amount of 3 μg of RNA of each sample was used for library construction, and ribosomal RNA was removed by Epicentre Ribo-zero rRNA Removal Kit (Epicentre, WI, USA). Sequencing libraries were prepared using the rRNA-depleted RNA by NEBNext Ultra Directional RNA Library Prep Kit for Illumina (NEB, MA, USA) following manufacturer’s recommendations. The library was quantified using the Qubit 2.0 Fluorometer (Thermo Scientific, MA, USA) and diluted into 1 ng/μL, and the library quality was evaluated on the Agilent Bioanalyzer 2100 system (Agilent Technologies, CA, USA). Clustering of the index-coded samples was conducted on a cBot Cluster Generation System using TruSeq SR Cluster Kit v3-cBot-HS (Illumia, CA, USA) following the manufacturer’s guidelines. The library preparations were then sequenced on the Illumina Hiseq. 2500 platform and 150 bp paired-end reads were produced.

### Transcripts expression quantification

Paired-end clean reads of mRNA sequencing (Table 2) were aligned to the Sscrofa11.1 genome assembly (https://www.ncbi.nlm.nih.gov/genome/?term=pig) using HISAT2^14^. More than 93% of the clean reads of each sample were mapped to the reference genome (Table 2). Read numbers mapped to each gene were counted using featureCounts^15^. The FPKM (expected number of Fragments Per Kilobase of transcript sequence per Millions base pairs sequenced) value of each gene was determined by the length of the gene and read counts mapped to this gene and used for estimating gene expression levels^16^. Gene expression data have been uploaded in Figshare^17^.

For miRNA clean reads, length filter was first processed for all samples (Table 1). The filtered reads were then mapped to the Sscrofa11.1 genome assembly using Bowtie^18^ without mismatch to analyze their expression and distribution (Table 2). Mapped small RNA tags were utilized to identify known miRNAs using miRDeep2^19^ based on miRBase 22 (http://www.mirbase.org/). Custom scripts were used to quantify the miRNA counts and base bias on the first position of identified miRNA with certain length. For novel miRNA prediction, miREvo^20^ and miRDeep2^19^ were integrated to predict novel miRNAs by exploring the second structure, Dicer cleavage site, and minimum free energy of the unannotated small RNA tags. miRNA expression levels were normalized as follows: normalized expression = mapped read count × 10^6^/library size^21^. Target gene prediction of miRNAs was performed using miRanda^22^. The expression levels of known miRNAs and novel miRNAs, and the predicted target genes are available at Figshare^17^.

The paired-end clean reads of lncRNA sequencing (Table 2) were aligned to the Sscrofa11.1 genome assembly using HISAT2^14^. The mapped reads of each sample were assembled using StringTie^23^ via a reference-based method. FPKM of lncRNAs in each sample was then calculated using StringTie^23^. The assembled transcripts were selected based on following criteria: number of exons ≥2; the length >200 bp nucleotides; non-overlap with the annotated exons in the reference genome. Four programs including Pfam-scan (v1.3)^24^, CPC2 (v0.1)^25^, PhyloCSF (v20121028)^26^, and CNCI (v2)^27^ with default parameters were used to assess the coding potential of transcripts. Transcripts predicted with coding potential by any of the four tools were removed, and those without coding potential were considered as candidate lncRNAs. Prediction of lncRNA-mRNA co-location networks was conducted with the parameters of upstream and downstream 100 kb of the location of lncRNAs. LncRNA-mRNA co-expression networks were predicted with R function “cor.test”, and mRNAs with absolute value of the correlation coefficient greater than 0.95 were retained. The expression levels of lncRNAs, lncRNA-mRNA co-location networks, and lncRNA-mRNA co-expression networks are available at Figshare^17^.

### Differential expression analysis

**Fig. 2:**
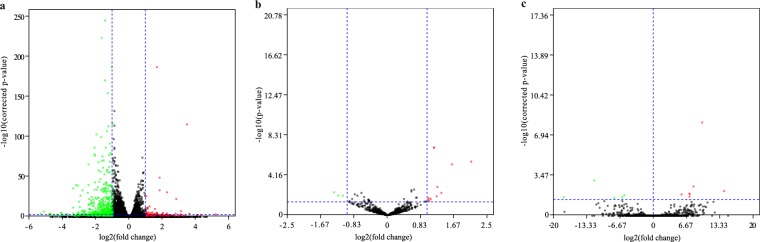
Volcano plot of differential expression profiles between ZEA-treated and control groups. (**a**) Differential expression of mRNAs. (**b**) Differential expression of miRNAs. (**c**) Differential expression of lncRNAs. Green dots represent significant down-expression, and red dots represent significant up-expression.

Following expression quantification, differential expression analyses of miRNAs, lncRNAs, and mRNAs between ZEA-treated and control groups were performed using DESeq. 2^28^. Benjamini and Hochberg’s method was applied to correct the resulting P-values for controlling false discovery rate. The mRNAs with a corrected P-value < 0.05 and |log2 fold change| ≥ 1 were defined as differentially expressed (Fig. 2a). The miRNAs with |log2 fold change| ≥ 1 and a P-value < 0.05 were defined as differential expression miRNAs (Fig. 2b). The lncRNAs with a corrected P-value < 0.05 were defined as differential expression lncRNAs (Fig. 2c). The differential expression data of miRNA, lncRNAs, and mRNAs are available at Figshare^17^.

### Library construction for ATAC-seq

ATAC-seq was conducted according to the protocols previously reported^29^. In brief, the nuclei were extracted and resuspended in the Tn5 transposase reaction mix. The transposition reaction was incubated at 37 °C for 30 min. Post transposition, the equimolar adapter1 and adapter 2 were added, and then PCR was performed to amplify the library. The library was purified with the AMPure beads and measured with Qubit 2.0 Fluorometer for quality assessment (Thermo Scientific, MA, USA). Clustering of the index-coded samples was performed on a cBot Cluster Generation System using TruSeq SR Cluster Kit v3-cBot-HS (Illumia, CA, USA) following the manufacturer’s instructions. The library preparations were sequenced on the Illumina Hiseq. 2500 platform by Novogene Bioinformatics Institute (Novogene, Beijing, China) and 150 bp paired-end reads were generated.

### ATAC-seq data analysis

**Fig. 3:**
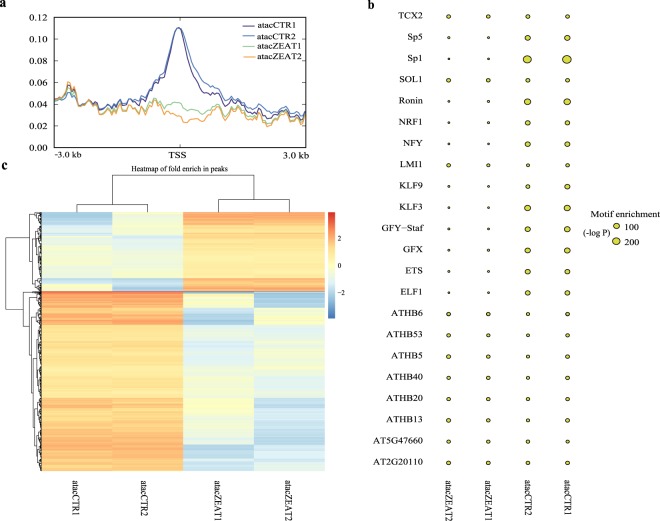
Reads distribution analysis and peak calling of ATAC-seq data. (**a**) Read density within 3 kb upstream and 3 kb downstream of the transcription start site. (**b**) Motif enrichment differences of known transcription factors in ZEA-treated and control samples. (**c**) Hierarchical clustering of peak enrichment patterns between ZEA-treated and control groups. TSS: transcription start site.

Paired-end clean reads (Table 3) were aligned to the Sscrofa11.1 genome assembly using BWA^30^ with default parameters. Read density (Fig. 3a) within 3 kb upstream and 3 kb downstream of the transcription start site was calculated using coumputeMatrix of DeepTools^31^. Peak calling was then performed using MACS2^32^. All reads were shifted towards the 3′ direction to the length of insert fragments, and the dynamic λ of each 200 bp sliding window was calculated. P values of each window were calculated based on the Poisson distribution and corrected using the false discovery rate method. The regions with a corrected P-value < 0.05 were defined as peaks, and the peaks have been submitted to Figshare^17^. The Homer software suite^33^ was utilized to recognize motif sequence in the 250 bp upstream and 250 bp downstream of the peak summits. Motif sequences were matched to the known motifs of transcription factors (Fig. 3b). The distance of peak summits to the nearest transcription start site and corresponding genes were analyzed using the PeakAnalyzer tool^34^. Differential peaks between ZEA-treated and control groups were identified by calculating the ratio of fold rich between the two groups. Peaks with |log2 fold rich ratio| ≥ 1 were defined as differential peaks. Hierarchical clustering analysis was performed to display the enrichment pattern of peaks in the two groups (Fig. 3c). Differential peaks between the two groups have been submitted to Figshare^17^.

## Data Records

The sequencing data of all experimental samples in the fastq format have been submitted to the Sequence Read Archive of NCBI under the accession number SRP218038^35^. The files of gene expression level and differential expression data between the two groups have been deposited in Figshare^17^.

## Technical Validation

### RNA quality control

RNA degradation and contamination was monitored on 1% agarose gels. The concentration and integrity of RNA samples were measured using the Qubit Fluorometer (Thermo Scientific, MA, USA) and Agilent 2100 Bioanalyzer platform (Agilent Technologies, CA, USA). Samples with rRNA ratio (28S/18S) ≥ 1.9 and RNA integrity number ≥8 were subjected to sequencing library construction.

### Quality validation and analyses

**Fig. 4:**
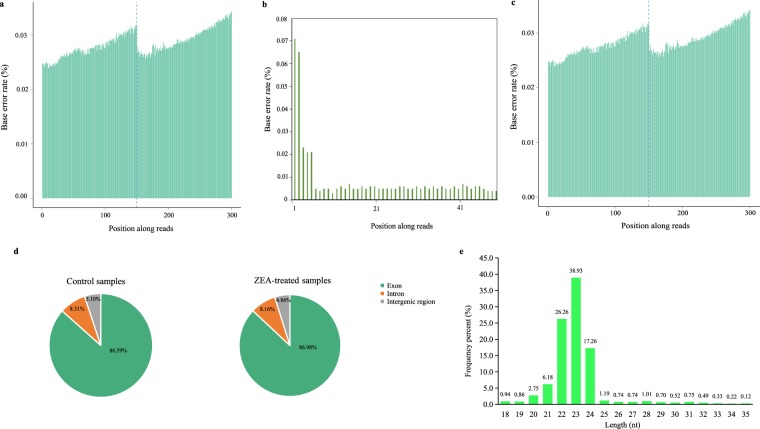
Quality assessment of mRNA, miRNA, and lncRNA sequencing data. (**a**) Error rate distribution along mRNA sequencing reads. (**b**) Error rate distribution along miRNA sequencing reads. (**c**) Error rate distribution along lncRNA sequencing reads. (**d**) Read distribution in genomic contexts of exon, intron, and intergenic regions. (**e**) Length distribution of mapped miRNA sequencing reads.

**Fig. 5:**
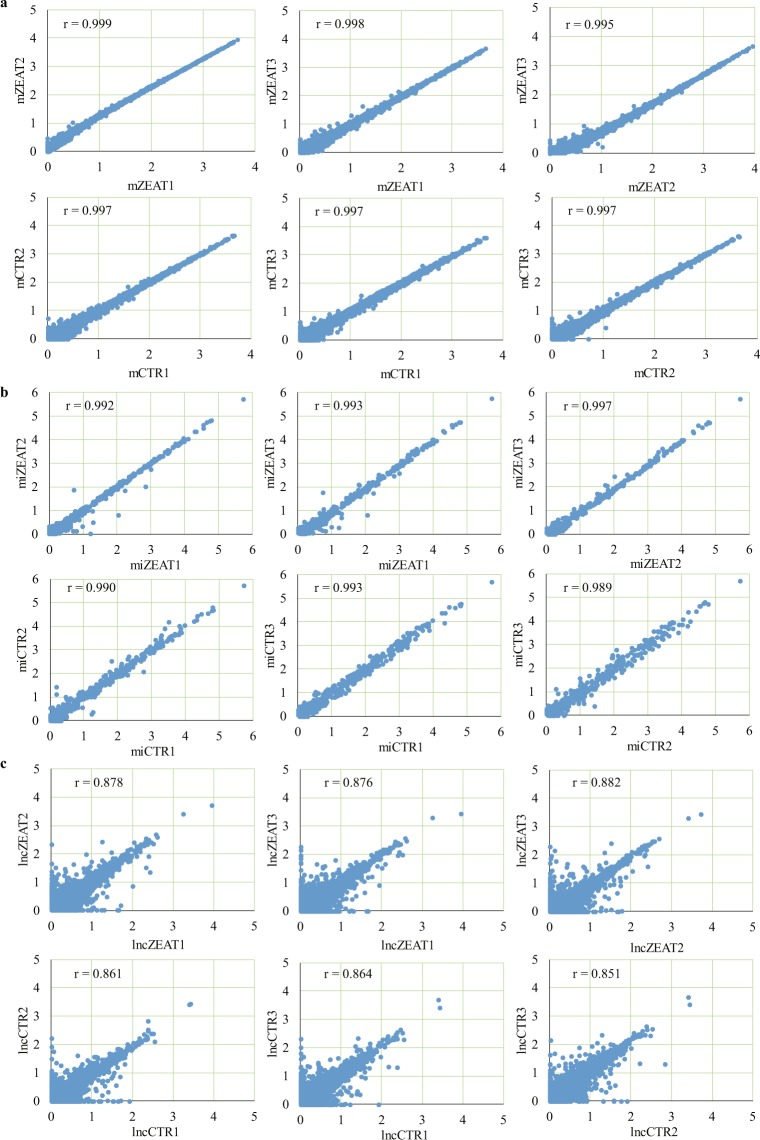
Pearson correlation analysis of experimental samples within ZEA-treated and control groups. (**a**) Pearson correlation between samples used for mRNA expression analysis. (**b**) Pearson correlation between samples used for miRNA expression analysis. (**c**) Pearson correlation between samples used for lncRNA expression analysis.

We examined the error rate of mRNA (Fig. 4a), miRNA (Fig. 4b), and lncRNA (Fig. 4c) read sequence and found high-quality sequences across all read bases. Raw sequencing data of mRNA, miRNA, and lncRNA (Table 2) were filtered to remove the reads with 5′ adapter contaminants, without 3′ adapter or the insert tag, with the proportion of N base greater than 10%, with poly A/T/G/C, and low quality reads (proportion of the bases with Qphred < = 20 greater than 30% of the total read bases) using FastQC^36^. All samples produced >97% clean reads after quality control, and >90% of clean reads were mapped to the reference genome (Table 2). In parallel, Q20, Q30, and GC content of the clean data were calculated (Table 2). These analyses indicated the high-quality of library construction and sequencing data of experimental samples. Genomic distribution analysis showed that on average 86.59% of the mapped reads of control samples and 86.98% of the mapped reads of ZEA-treated samples were mapped to exons (Fig. 4d), suggesting the efficient reflection of genome-wide gene expressions. Length distribution analysis of miRNA read sequence showed that most of the reads (88.6%) were in the length of 21~24 nt (Fig. 4e), which was consistent with the biological features of small RNAs. Pearson correlation analysis was performed to further examine the reproducibility of biological replicates in different groups. Correlation coefficients of mRNA sequencing replicates within ZEA-treated and control groups were greater than 0.99 (Fig. 5a). Correlation coefficients of miRNA sequencing replicates within the two groups were greater than 0.98 (Fig. 5b), and those of lncRNA sequencing replicates within the two groups were greater than 0.85 (Fig. 5c).

**Fig. 6:**
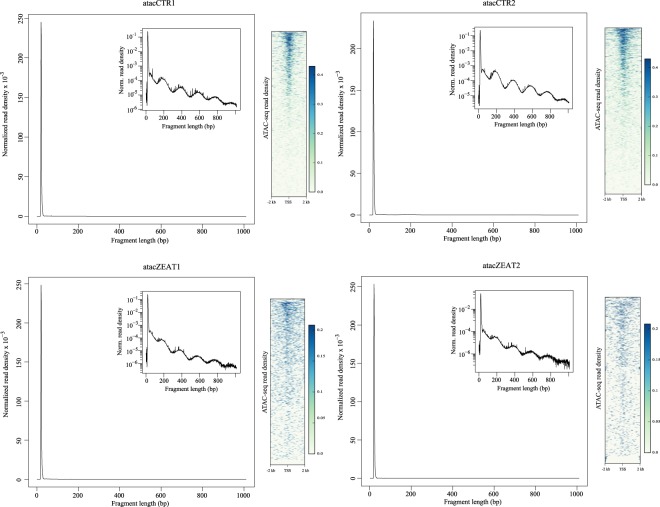
ATAC-seq data quality control metrics of fragment size distribution and sequencing read enrichment around transcription start sites. TSS: transcription start site.

**Fig. 7:**
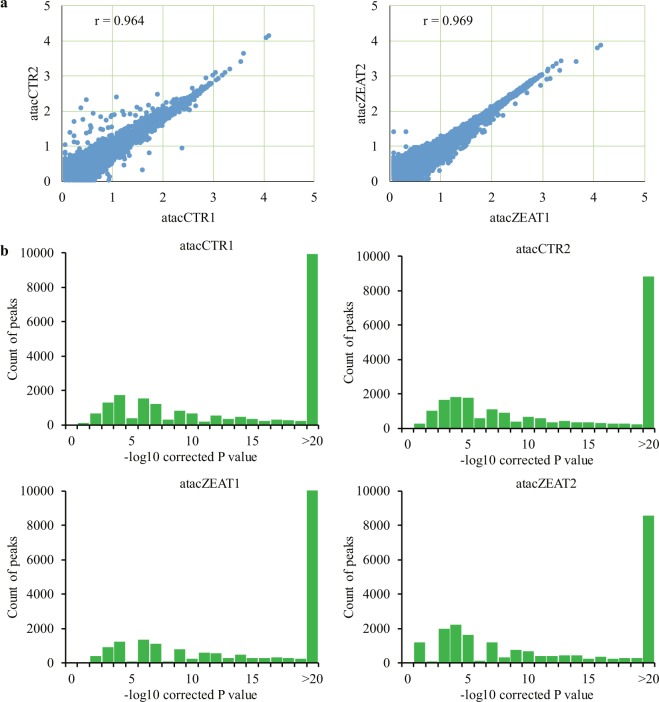
Quality assessment of ATAC-seq data. (**a**) Pearson correlation of experimental samples within ZEA-treated and control groups. (**b**) Peak score (−log10 (corrected P value)) distribution of each experimental sample.

For ATAC-seq data, raw reads (Table 3) were first trimmed using Skewer^37^ to remove the reads with sequencing adaptors, with proportion of N base greater than 10%, low quality reads (proportion of the bases with Qphred < = 20 greater than 30% of the total reads bases), and the reads with the length smaller than 18 nt after trimming. To ensure the reliability of read mapping, reads with mapping quality > 13 and properly paired reads were retained for subsequent analysis. The size distribution of sequenced fragments displayed clear periodicity, and the regions around transcription start sites were enriched for ATAC-seq reads (Fig. 6). The two standard quality metrics demonstrated the ATAC-seq data quality to capture the accessible chromatin regions (Fig. 6). Moreover, the Pearson correlation coefficients are 0.969 of ZEA-treated replicates and 0.964 of control replicates (Fig. 7a), indicating high reproducibility of accessible chromatin regions between replicates within the two groups. We identified peaks by using the MACS2 program^32^. Peak scores (–log10 (corrected P value)) were calculated and most of the peaks showed a peak score >20 (Fig. 7b), indicating the high reliability of peak calling.

## Data Availability

HISAT2: http://ccb.jhu.edu/software/hisat2/index.shtml. Version: 2.0.5. Parameters: –rna-strandness RF. Bowtie: http://bowtie-bio.sourceforge.net/bowtie2/index.shtml. Version: 0.12.9. Parameters: -v 0 –k 1. miRDeep2: https://github.com/rajewsky-lab/mirdeep2. Version: 2.0.0.8. Parameters: quantifier.pl -p -m -r -y -g 0 -T 10. miREvo: http://evolution.sysu.edu.cn/software/mirevo.htm. Version: 1.1. Parameters: -i -r -M -m -k -p 10 -g 50000. miRanda: http://miranda.org.uk/. Version: 2.042. Parameters: -sc 140 -en -10 –scale 4 -strict -out. StringTie: http://ccb.jhu.edu/software/stringtie/. Version: 1.3.1. Parameters: default. BWA: http://bio-bwa.sourceforge.net/. Version: 0.7.12. Parameters: -T 25 -k 18. DeepTools Version: 3.0.2. Parameters: –cor Method Pearson. MACS2: http://liulab.dfci.harvard.edu/MACS/. Version: 2.1.2. Parameters: -q 0.05–call-summits –nomodel –shift -100 –extsize 200 –keep-dup all. Homer: http://homer.ucsd.edu/homer/. Version: 4.9.1. Parameters: -gc –len 8, 10, 12, 14.
